# A Rare Glimpse into the Past of the Anthrax Pathogen *Bacillus anthracis*

**DOI:** 10.3390/microorganisms8020298

**Published:** 2020-02-21

**Authors:** Peter Braun, Mandy Knüpfer, Markus Antwerpen, Dagmar Triebel, Gregor Grass

**Affiliations:** 1 Bundeswehr Institute of Microbiology (IMB), 80937 Munich, Germany; peter3braun@bundeswehr.org (P.B.); mandyknuepfer@bundeswehr.org (M.K.); markusantwerpen@bundeswehr.org (M.A.); 2 Bavarian Natural History Collections (SNSB—Botanische Staatssammlung München), 80638 Munich, Germany; triebel@snsb.de

**Keywords:** anthrax, *Bacillus anthracis*, historic specimen, genome-sequencing, phylogeny

## Abstract

The bacterium *Bacillus anthracis* is the causative agent of the zoonotic disease anthrax. While genomics of extant *B. anthracis* isolates established in-depth phylogenomic relationships, there is scarce information on the historic genomics of the pathogen. Here, we characterized the oldest documented *B. anthracis* specimen. The inactive 142-year-old material originated from a bovine diseased in Chemnitz (Germany) in 1878 and is contemporary with the seminal studies of Robert Koch on *B. anthracis*. A specifically developed isolation method yielded high-quality DNA from this specimen for genomic sequencing. The bacterial chromosome featuring 242 unique base-characters placed it into a major phylogenetic clade of *B. anthracis* (B.Branch CNEVA), which is typical for central Europe today. Our results support the notion that the CNEVA-clade represents part of the indigenous genetic lineage of *B. anthracis* in this part of Europe. This work emphasizes the value of historic specimens as precious resources for reconstructing the past phylogeny of the anthrax pathogen.

## 1. Introduction

For notorious pathogens such as *Yersinia pestis* (plague) or *Mycobacterium tuberculosis* (tuberculosis), there is ample information on the historic phylogeography of the pathogens from human remains. For instance, the oldest molecular evidence for historic plague from Sweden is 4900 years old [1]. In contrast, for *Bacillus anthracis*, the bacterium causing the zoonotic disease anthrax, our knowledge on its historic phylogeny hardly reaches back more than a hundred years. Though Louis Pasteur performed his famous public anthrax vaccination experiment in 1881, the Collection de l’Institut Pasteur (CIP) only initiated the collection of bacterial strains in 1892 [2]. These strains, however, have been cultured ever since, and thus likely have accumulated recent genetic changes. Conversely, dead specimens or bacteria from human remains are evolutionary inert.

Anthrax has plagued humans and both wild and domestic animals for hundreds—possibly many thousands-of years [3]. Even today, the pathogen is rampant in numerous countries on all continents except Antarctica [4]. Only a few countries have managed, through governmental and institutional vaccination campaigns, disease reporting and safe cadaver disposal programs to all but eradicate anthrax outbreaks today. Even Germany, which has seen very limited minor animal outbreaks recently in 2009, 2012, and 2014 [5], had been stricken by the disease only a century ago. From 1912 to 1932 (no data for 1926), 2518 people became infected, of which 431 died. Within the same time period, about 90,000 domestic animals fell from the disease [6]. Within that time occurred the infamous “Baron von Rosen espionage incident” involving sugar lumps allegedly laced with the anthrax pathogen aimed at sabotaging allied horse-powered war-support-lines in 1917 [7,8]. While there is a large number of live historical *B. anthracis* isolates in culture, the oldest one mentioned in literature is from approx. 1890 [9] but lacks associated metadata. Thus, its origin remains elusive. Finally, *B. anthracis* has recently been isolated from excavation sites at the permafrost zone of northern Russia [10]. If these isolates, however, indeed originated from thousands-of-years-old cadavers devoid of any vegetative episodes of germinating spores and re-sporulating bacteria remains debatable. Also, it cannot be excluded that spores from younger outbreaks have contaminated deeper, older soil horizons. Nevertheless, with large permafrost areas thawing, we might see reemerging anthrax in high latitudes [10].

Historic, fixed specimens of *B. anthracis* on glass slides have attracted surprisingly little interest as valuable means for expanding our knowledge on the anthrax pathogen diversity. Published works comprise information from relatively young samples only: a couple of years old from Jordan [11], up to 30 years old from Zambia [12], or about 35-year-old paraffin-embedded samples from the former Soviet Union [13].

Thus, to date, there is no described bona fide historical genome of *B. anthracis*. In this report, we characterized the oldest documented *B. anthracis* genome from a 142-year-old historic microscopic slide.

## 2. Materials and Methods

### 2.1. B. anthracis Strains, Growth Conditions, and Extraction of DNA from Inactivated Culture Material

All strains (chromosomes) used in this study are listed in Supplementary Table S1. *B. anthracis* cultures from our strain collections were cultivated in our biosafety level 3 laboratory on blood agar and then chemically inactivated before further use [14]. DNA was isolated using MasterPure™ Gram Positive DNA Purification kit (Lucigen, Middleton, WI, USA) and DNA concentrations were quantified using the Qubit dsDNA HS Assay Kit (Thermo Fisher Scientific, Dreieich, Germany) according to the manufacturers’ protocols. DNA preparations were stored at −20 °C until further use.

### 2.2. Microscopic Evaluation of the Historical B. anthracis Specimen

The original glass slide featuring a *B. anthracis* blood smear was carefully unwrapped from cover envelopes and examined by phase contrast microscopy (630-fold magnification) using a Leica DMi8 inverted light microscope (Leica Microsystems, Wetzlar, Germany).

### 2.3. DNA Extraction from the Historical B. anthracis Blood Smear

All manipulations related to sample Chemnitz 1878 (M-0290509 published in 1879 as no. 700 of the exsiccatae series [15]) were conducted in a laboratory not previously utilized to handle *B. anthracis* DNA in order to avoid contamination. A section of the blood smear was carefully removed using a sterile swab (Copan Nylon Floq Swab, Hain Life Science, Nehren, Germany) moistened with 100 µL of sterile phosphate-buffered saline by slowly rotating the swab and sampling the surface in a zig-zag-like movement. The swab was air-dried for 15 min under a laminar air flow. The swab head was then cut off and placed into a 2-mL microcentrifuge tube. DNA was extracted using MasterPure™ Gram Positive DNA Purification kit (Lucigen, Middleton, WI, USA), using a modified protocol. Briefly, 150 µL of TE buffer containing 1250 U of Ready-Lyse lysozyme solution was added directly onto the swab head and incubated at 37 °C for 60 min. To this, 1 μL of proteinase K (50 μg/μL) diluted into 150 μL of Gram-positive lysis solution was added. The tube was then incubated at 65 °C for 15 min at 900 rpm, briefly vortexed every 5 min. After placing the sample on ice for 5 min, the liquid and the swab head were transferred to a QIAshredder spin column (Qiagen, Hilden, Germany) and centrifuged at 14,000 rpm for 2 min. The swab head was discarded and 175 µL of ‘MPC’ Protein Precipitation Reagent was added to the flow-through. After vortexing, the debris was pelleted by centrifugation at 4 °C for 10 min at 14,000 rpm in a microcentrifuge. The supernatant was transferred to a clean microcentrifuge tube. To this, 5 µL of Roti-Pink (Carl Roth, Karlsruhe, Germany), 10 µL of glycogen solution (5 mg/mL, Carl Roth, Karlsruhe, Germany), and 500 µL of isopropanol (Carl Roth, Karlsruhe, Germany) was added and gently mixed by inverting the tube 30–40 times. DNA was pelleted by centrifugation at 4 °C for 10 min at 14,000 rpm in a microcentrifuge. The DNA pellet was washed twice with 200 µL 70% ethanol. After removing all of the residual ethanol, the DNA was resuspended in 50 µL TE buffer and stored at −20 °C until further use. 

### 2.4. Whole Genome Sequencing and Data Analysis—Single Nucleotide Polymorphism Calling

From total DNA, an Illumina-compatible library was prepared (NEBNext^®^ Ultra™ II DNA Library Prep Kit, NEB, Frankfurt am Main, Germany) and sequenced on a MiSeq instrument (Illumina, San Diego, CA, USA) using MiSeq V3-chemistry. High-quality paired-end reads were assembled de novo using an in-house script based on SPAdes assembler [16] and Pilon [17] for correcting genome-assembly. In order to exclude age-related DNA-sequencing artifacts and to avoid incorrect conclusions, the genome sequence was curated manually as follows: First, obtained scaffolds (software BWA-SW [18]) were mapped to *B. anthracis* strain BF-1, a close genetic neighbor. Regions not covered by any reads were excluded from the consensus sequence. Second, BWA-mem was used to remap reads to the ordered contigs. Third, for eliminating ambiguous base positions, mpileup (software-package SAMtools [19]) was used with standard parameters. All ambiguous positions (*n* = 9326; i.e., 0.17% of the chromosome) were masked with “N” in the corresponding consensus-fasta-file. This final curated sequence was used for further comparative analyses. All data generated and analyzed during this study are included in this published article, its supplementary information files, or are publicly available in the NCBI Sequence Read Archive (SRA) repository (Bioproject PRJNA309927).

For multiple chromosome-wide SNP-comparison of *B. anthracis*, the Parsnp tool (Harvest Suite) was used [20]. For this, representative *B. anthracis* chromosomes from public databases (Supplementary Table S1) and newly sequenced chromosomes Chemnitz 1878, Tyrol 3520 and 6282 were aligned (Parsnp parameters -c -e -u -C 1000) using *B. anthracis* Ames Ancestor reference chromosome (NC_007530) as phylogenetic outgroup.

Called SNPs were extracted into a multi-isolate-vcf file using the HarvestTools (version 1.0) from the same software suite [20]. To enhance data quality, closely adjacent SNPs with a distance of less than 10 bp as well as positions harboring undefined nucleotides (“N”) were removed. This curated vcf-file was used as an input file in the HarvestTools to compile a FASTA-file comprising the concatenated SNPs of the investigated chromosome set as multiple-sequence alignment. 

This concatenated sequence information was used to infer and analyze a maximum likelihood tree-based phylogeny in MEGA 7 [21,22]. SNPs found within the analyzed *B. anthracis* chromosomes are summarized in Supplementary Table S2. A minimum spanning tree was computed in BioNumerics 6.6 (Applied Maths, Sint-Martens-Latem, Belgium) from the vcf SNP-file (in binary format) as input and manually edited for style.

## 3. Results and Discussion

### 3.1. Characterization of a Glass Slide Specimen Labeled B. anthracis from 1878.

**Figure 1:**
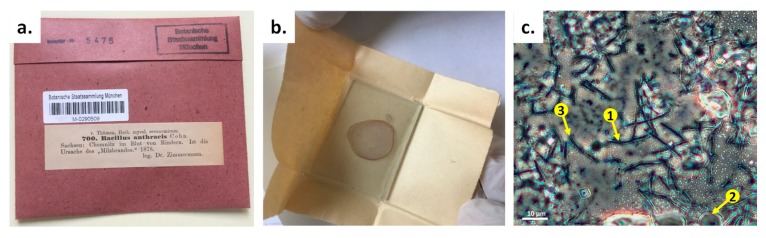
Initial characterization of a *B. anthracis* specimen from 1878. (**a**) The specimen with original label (translated from German: “Saxonia: Chemnitz in the blood of bovines. It is the source of ‘anthrax’”). (**b**) Unpacking of the specimen, which was a glass slide paper-wrapped without any further glass cover. The red color from blood cells can still be identified. (**c**) Microscopic (phase-contrast) examination of the specimen. Arrow #1 indicates rod-like structures, likely *B. anthracis* cells; arrow #2 indicates possible bovine blood cells; and arrow #3 indicates structures that resemble nascent endospores.

The 142-year-old microscopic slide featured an infected blood specimen from the Bavarian Natural History Collections SNSB—*Botanische Staatssammlung München* (Germany). It was rediscovered during an inventory in 2018. The slide envelope was orderly labeled, providing information on the responsible veterinarian and the time and geographic location of the diseased bovine from which the blood smear was taken (Figure 1a). A veterinarian named Dr. O. E. R. Zimmermann prepared the smear from infected bovine blood on a glass slide in 1878 (Figure 1b), only two years after Robert Koch started systematic research on *B. anthracis*. Careful microscopic documentation of the specimen indicated rod-like structures among likely dried-up bovine blood cells and even possible nascent spores, supporting the claim *B. anthracis* was indeed possibly present (Figure 1c).

### 3.2. DNA Isolation from Specimen Chemnitz 1878 and Genome Sequencing Yielded a B. anthracis Genome Typical for Central Europe

Using an improved swab-based extraction method specifically developed for this need, we were able to isolate DNA from the historic specimen. Unexpectedly, the quality and quantity of the extracted DNA was sufficient for PCR and subsequent whole-genome sequencing. Thus, 2 × 21,106,786 reads were generated. Of the six Gb obtained, only about 2% (117,288,692 bases) were *B. anthracis*. The remaining 98% reads were of other bacterial (e.g., *Cutibacterium* sp.), bovine, or human (6,590,587 reads; 31%) origin. The latter likely reflecting repeated contact with museum staff during the last 100+ years of storage, because the slide was not glass-covered.

**Figure 2:**
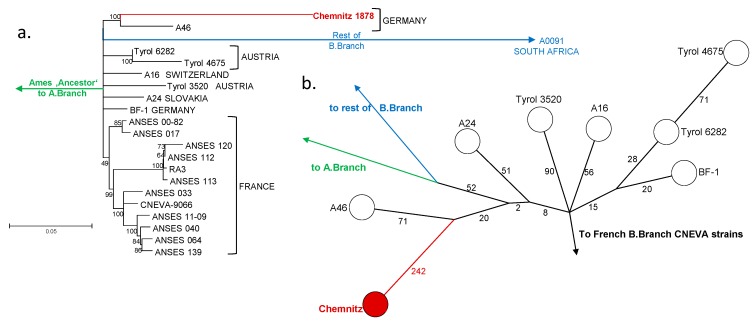
Phylogenetic placement of *B. anthracis* specimen Chemnitz 1878. (**a**) Rooted maximum likelihood tree derived from chromosomal Single Nucleotide Polymorphisms of Chemnitz 1878 and representative relatives (2450 chromosomal SNPs in total; bootstrap confidence-values based on 500 permutations). Isolate names and countries of origin are indicated at branch termini. The tree is rooted to the *B. anthracis* reference strain Ames ‘Ancestor’, which belongs to the A.Br.Ames clade. (**b**) Minimum spanning tree based on chromosomal SNPs showing strain Chemnitz 1878 alongside its closest relatives. Numbers next to branch-lines indicate SNPs separating nodes or strains.

Genomic in silico analysis revealed that the chromosomal *B. anthracis* PCR-marker *dhp61* was present as well as both virulence plasmids pXO1 and pXO2. Canonical SNP-typing phylogenetically placed the historic genome, which we named Chemnitz 1878, within the B.Br.CNEVA clade of *B. anthracis* [23]. Next, we inferred the phylogenetic placement of the Chemnitz 1878 chromosome within the B.Br.CNEVA clade of *B. anthracis* (Figure 2a). 

The closest living relative was strain A46, isolated from a pig near Stuttgart (Germany), with a distance of 313 SNPs, though a more distant relative, cattle isolate BF-1 from Bavaria, was at 307 SNPs distance (Figure 2b). Isolates from central Europe (Austria, Switzerland, and Slovakia) were grouped to the same lineage). From these, strain Tyrol 4675 featured the most SNP differences to strain Chemnitz 1878 (386 SNPs; Figure 2b). B.Br.CNEVA strains from France grouped phylogenetically further away, forming several distinct sub-clusters within CNEVA canSNP group (Figure 2a). Notably, with new B.Br.CNEVA-genomes now available, there is a significant polytomy right at the base of the B.Br.CNEVA lineage (Figure 2a), contrary to what was reported before based on more limited information [9].

While German *B. anthracis* strain collections feature a broad diversity of eight out of the twelve original *B. anthracis* canSNP-groups [23], recent data suggests B-branch isolates constituting the autochthonous population of the anthrax pathogen in countries from central [9] and northern [24] Europe, as well as from northwestern Asia (Russian Federation) [10]. Thus, from a bioforensics point of view, the isolation of a B.Br.CNEVA-type *B. anthracis* strain from a future outbreak would raise fewer concerns than would an isolate from a canSNP-group typical for a non-European origin.

## 4. Conclusions

This work emphasizes that historic specimen slides in mycological collections of herbaria, museums, and alike may constitute invaluable sources for reconstructing the historic phylogeny of the anthrax pathogen in countries in which the disease is all but eradicated today. Making this approach more broadly known would likely also avoid such mishaps (from a scientific point of view) as the one at the Chrysler Herbarium at Rutgers University in 2016 (https://news.illinois.edu/view/6367/350255). During a digitization project of historical samples, a 121-year-old specimen, an envelope labeled *B. anthracis*, was unearthed. Unfortunately, the envelope’s content had been destroyed years before without further characterization.

## Supplementary Materials

The following are available online at https://www.mdpi.com/2076-2607/8/2/298/s1: Table S1: Genome sequences accession numbers of newly sequenced and additional *B. anthracis* strains from publicly available databases; Table S2: Chromosome-wide binary SNP matrix of all analyzed *B. anthracis* chromosomes.
